# Whole-Exome Sequencing Reveals a Missense Variant c.1612C>T (p.Arg538Cys) in the *BTD* Gene Leading to Neuromyelitis Optica Spectrum Disorder in Saudi Families

**DOI:** 10.3389/fped.2021.829251

**Published:** 2022-02-21

**Authors:** Muhammad Imran Naseer, Peter Natesan Pushparaj, Angham Abdulrahman Abdulkareem, Osama Y. Muthaffar

**Affiliations:** ^1^Center of Excellence in Genomic Medicine Research, King Abdulaziz University, Jeddah, Saudi Arabia; ^2^Department of Medical Laboratory Technology, Faculty of Applied Medical Sciences, King Abdulaziz University, Jeddah, Saudi Arabia; ^3^Center for Transdisciplinary Research, Department of Pharmacology, Saveetha Dental College and Hospitals, Saveetha Institute of Medical and Technical Sciences, Chennai, India; ^4^Department of Biochemistry, Faculty of Science, King Abdulaziz University, Jeddah, Saudi Arabia; ^5^Department of Pediatrics, Faculty of Medicine, King Abdulaziz University, Jeddah, Saudi Arabia

**Keywords:** biotin, biotinidase, vision loss, myelopathy, neuromyelitis optica, WES

## Abstract

Biotinidase deficiency is an autosomal recessive, multiple carboxylase deficiency usually associated with seizures, eczema, hypotonia, visual disturbances, hearing loss, and developmental delays. Only a handful of cases of biotinidase deficiency that had clinical features of neuromyelitis optica spectrum disorder have been reported in the literature. The case report study is about the clinical and genetic features of two pediatric patients from different families with biotinidase deficiency whose brain and spine MRI scans were suggestive of neuromyelitis optica. Neither child improved with immunotherapy. They come from a first-degree blood-related family. In both cases, a deficiency of the enzyme biotinidase was detected. The missense variant NM_001370658.1 (BTD):c.1612C>T (p.Arg538Cys) NM_000060.4 in exon 4 was identified by whole-exome sequencing. The identified sequence variation was validated using Sanger sequencing analysis. The intake of biotin resulted in clinical improvement. After a follow-up period of 12 months, the patient was gradually weaned from tracheostomy. His vision had improved significantly. He was able to walk and run independently. In conclusion, biotinidase deficiency is a rare and treatable cause of neuromyelitis optica. Early diagnosis can prevent poor clinical outcomes. Biotinidase enzyme levels should be considered as part of the examination algorithm for neuromyelitis optica spectrum disorder.

## Introduction

Biotinidase deficiency is caused by pathogenic variants in the *BTD* gene, located on chromosome 3p25.1. Low serum biotinidase activity is seen in these patients. The enzyme biotinidase cleaves biocytin into biotin as a coenzyme in carboxylation reactions. Low enzyme activity can be detected in serum by newborn screening. Multiple carboxylase deficiency (MCD) is a biotin-responsive disorder in which affected infants present with infections, skin rash, acute intermittent ataxia, and lactic acidosis (1). Similarly, one patient showed atrophy of the superior cerebellum, and three affected children from two unrelated families showed late-onset multiple carboxylase deficiency (2).

If diagnosed early, it is a potentially treatable metabolic disorder. The incidence of biotinidase deficiency is 1/60,000 individuals worldwide. However, it is believed to be higher in regions with high consanguinity such as Saudi Arabia. In Turkey, the incidence of biotinidase deficiency is estimated to be 1/7,116 individuals. Biotinidase deficiency can occur in early infancy, in childhood, and as a late consequence in adulthood. In infancy and childhood, biotinidase deficiency results in dermatitis, alopecia, hypotonia, developmental delay, seizures, optic atrophy, hearing loss, and respiratory problems (3). The late-onset form is more characterized by myelopathy and vision loss. Oral biotin supplementation can lead to dramatic clinical improvement, especially if diagnosed early.

Neuromyelitis optica spectrum disorder (NMOSD) is a spectrum of antibody-mediated demyelinating disorders of the central nervous system characterized by classic demyelinating changes in the spinal cord and optic nerve involvement. A partial response to initial therapy with steroids has already been reported in *BTD* mimicking NMOSD (4, 5). In these cases, the correct diagnosis was made only after presentation with a relapse of symptoms. The elevated lactate and alanine levels in the CSF (more than expected in NMOSD) indicated a possible metabolic cause (4, 6).

The classic MRI features of NMOSD are hyperintensities of the optic nerves, demyelination of the periaqueductal gray matter, hypothalamic involvement, and changes in the long segment of the spinal cord. The presence of aquaporin 4 antibodies (AQP4-Ab) supports the diagnosis. It can lead to severe motor disabilities and vision loss (7). Early diagnosis, aggressive immunotherapy, and long-term immunosuppression can improve the prognosis of such disease.

Biotinidase deficiency with a clinical picture mimicking NMOSD has not been reported in the Middle East. Few cases have been discussed in the literature on this topic. In this article, we discuss the clinical and genetic presentation of two pediatric cases.

## Results

### Clinical Details of the Families

#### Patient 1

**Figure 1:**
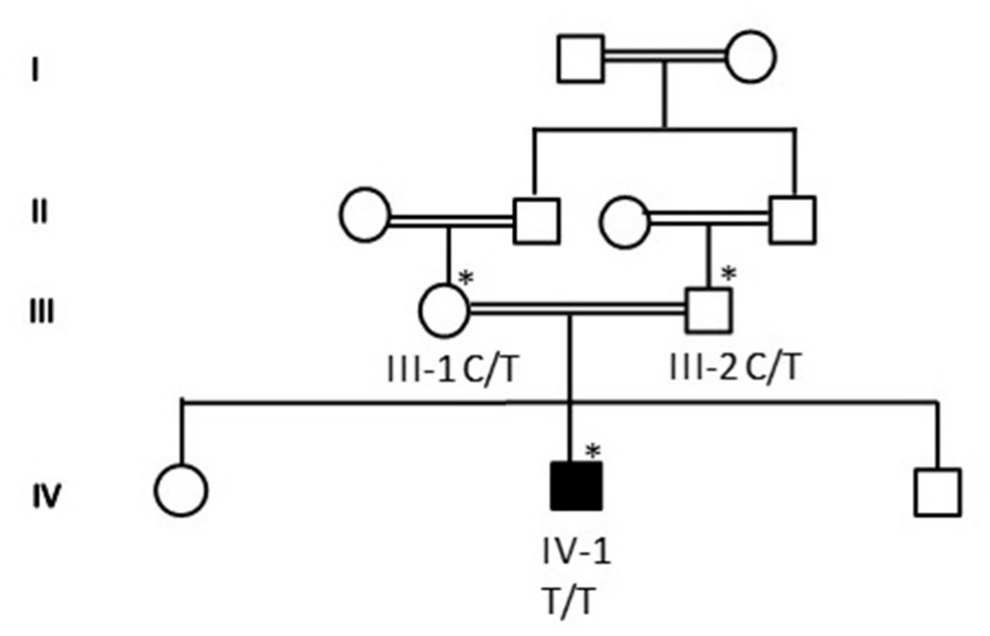
A detailed pedigree was drawn after obtaining information from the parents. The symbol * showed the available samples for the study.

Proband IV-4 from family 1 was a healthy child. He presented at the age of 4 years with fever and weakness of lower limbs. He had mild fever (38°C) and runny nose for 3 days. Then ascending lower limb weakness was gradually noted. No urinary or fecal incontinence was observed. He has no back pain, no paresthesias, and no history of trauma. The family denied any history of hearing impairment. His development was age appropriate. The parents are first cousins. The detailed family pedigree was drawn as shown in Figure 1.

The other two siblings are healthy. His clinical examination showed normal mental status. Visual acuity was 10/100 in both eyes. His strength was 4/5 in upper limbs and 1/5 in lower limbs. His deep tendon reflexes were decreased all over. He had no sensory deficit and normal skin examination.

**Figure 2:**
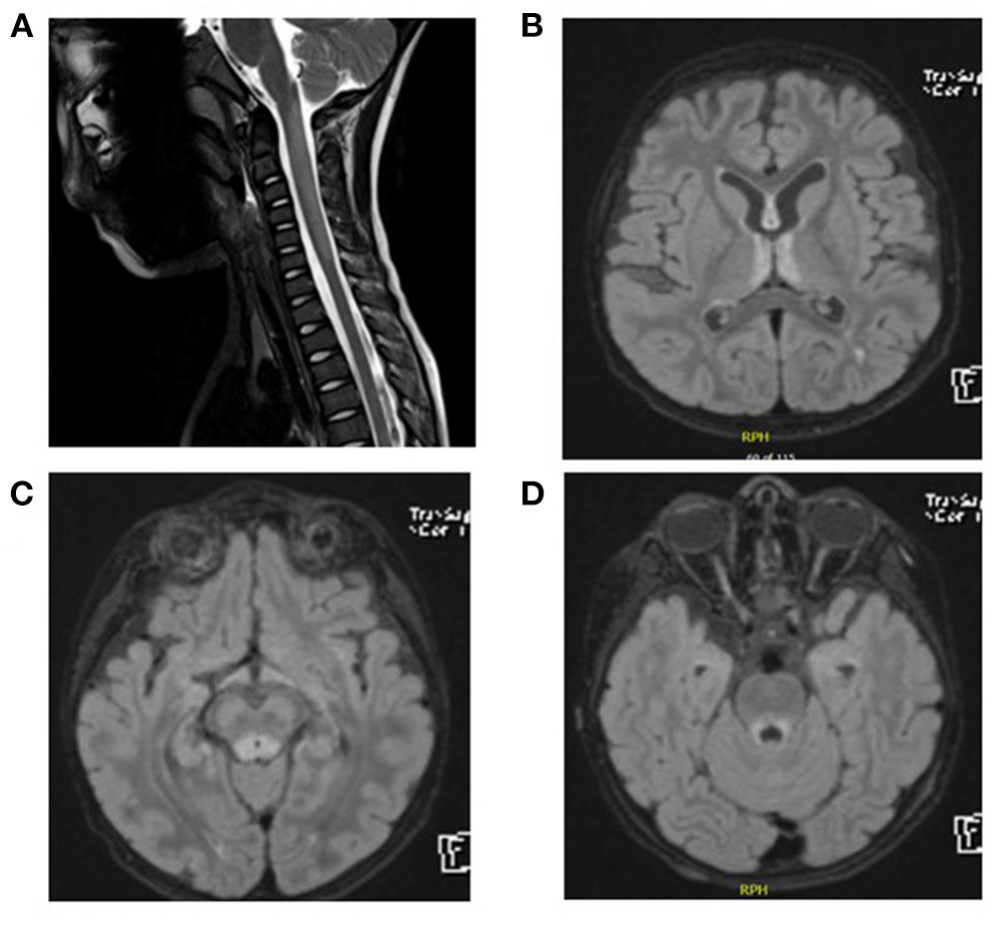
**(A)** Spinal MRI. Expansile central intramedullary abnormal high T2 signal intensity involving the upper cervical cord extending from the level of the cervicomedullary junction down to the level of the C5 vertebral body. **(B–D)** Brain MRI and FLAIR images. T2 signal hyperintensities seen on the medial thalami, dorsal aspect of the midbrain, and periaqueductal area of the midbrain.

CSF analysis showed negative culture including HSV1–2. CSF protein was 0.3 g/L (NR: 0.15–0.45 g/L), and the CSF glucose level was 55 mg/dl (NR: 45–75 mg/dl). The oligoclonal bands in the CSF were also negative. Thyroid function tests, anti-TPO, and anti-TG were normal. Anti-nuclear antibody profile (ANA) was normal. AQP4-Abs and anti-myelin oligodendrocyte glycoprotein antibodies (MOG) were negative. He received 5 days of intravenous immunoglobulin (IVIG) treatment and was discharged home. His ability to walk remained impaired for the next 3 months after IVIG treatment. He then presented with difficulty in swallowing and an inability to see objects clearly. An MRI of the brain and spine was performed and showed demyelination of the periaqueductal gray matter, hypothalamic involvement, and bilateral symmetric high T2 signal intensity of the spinal cord (Figures 2A–D). Serum AQP4-Ab was negative. A 5-day course of methylprednisolone pulse therapy and another course of IVIG were started. Over the next 4 months, his vision continued to decline, he became bedridden, and he presented to the emergency department with respiratory distress and aspiration pneumonia. He was then intubated for 2 weeks. The biotinidase level was low at 0.2 nmol/min/ml (reference range 4.4–12.5 nmol/min/ml). He was treated with biotin 20 mg/kg/day. Over the next 12 months, he was gradually weaned from tracheostomy. His vision has improved significantly. He can now walk and run independently.

#### Patient 2

He presented at the age of 3 years and 6 months. Previously, he was healthy until he developed weakness of the lower limbs and inability to walk, which developed gradually over 1 week. Gradually, he developed respiratory problems and was ventilated for 4 months. He received several courses of immunotherapy with no clinical benefit. He lost his vision and became ventilator dependent. He had no history of fever, no trauma, and no similar cases in his family. Parents are first cousins, and his sibling is healthy. On clinical examination, he was bedridden and on mechanical ventilator. His strength was 3/5 in the upper and lower extremities. He was hypotonic. Deep tendon reflexes were suppressed.

**Figure 3:**
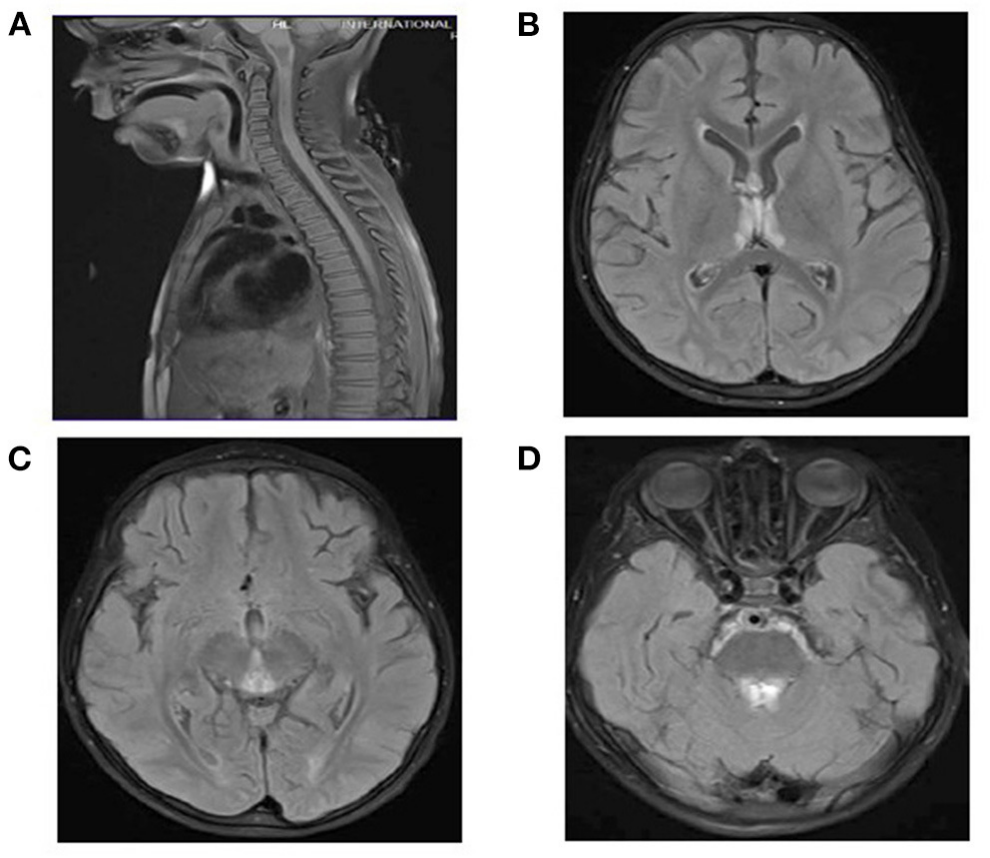
**(A)** Spinal MRI: Normal. **(B–D)** Brain MRI and FLAIR images. T2 signal hyperintensities seen on the medial thalami, dorsal aspect of the midbrain, and periaqueductal area of the midbrain.

CSF analysis revealed a negative culture including HSV1–2. White blood cell count in the CSF was six cells per cubic meter, and red blood cell count was one cell per cubic meter. Glucose and protein in CSF were within normal range. AQP4-Abs and anti-MOG were negative. MRI of the brain and spine showed similar findings to the other patient (Figures 3A–D). Serum biotinidase was low at <1 nmol/min/ml (reference range 5.1–11.9 nmol/min/ml). He was switched to biotin 20 mg/kg/day. After a follow-up period of 12 months, he was discharged home with a tracheostomy, his vision started to improve, and he is able to use a wheelchair.

### Whole-Exome Sequencing

**Figure 4:**
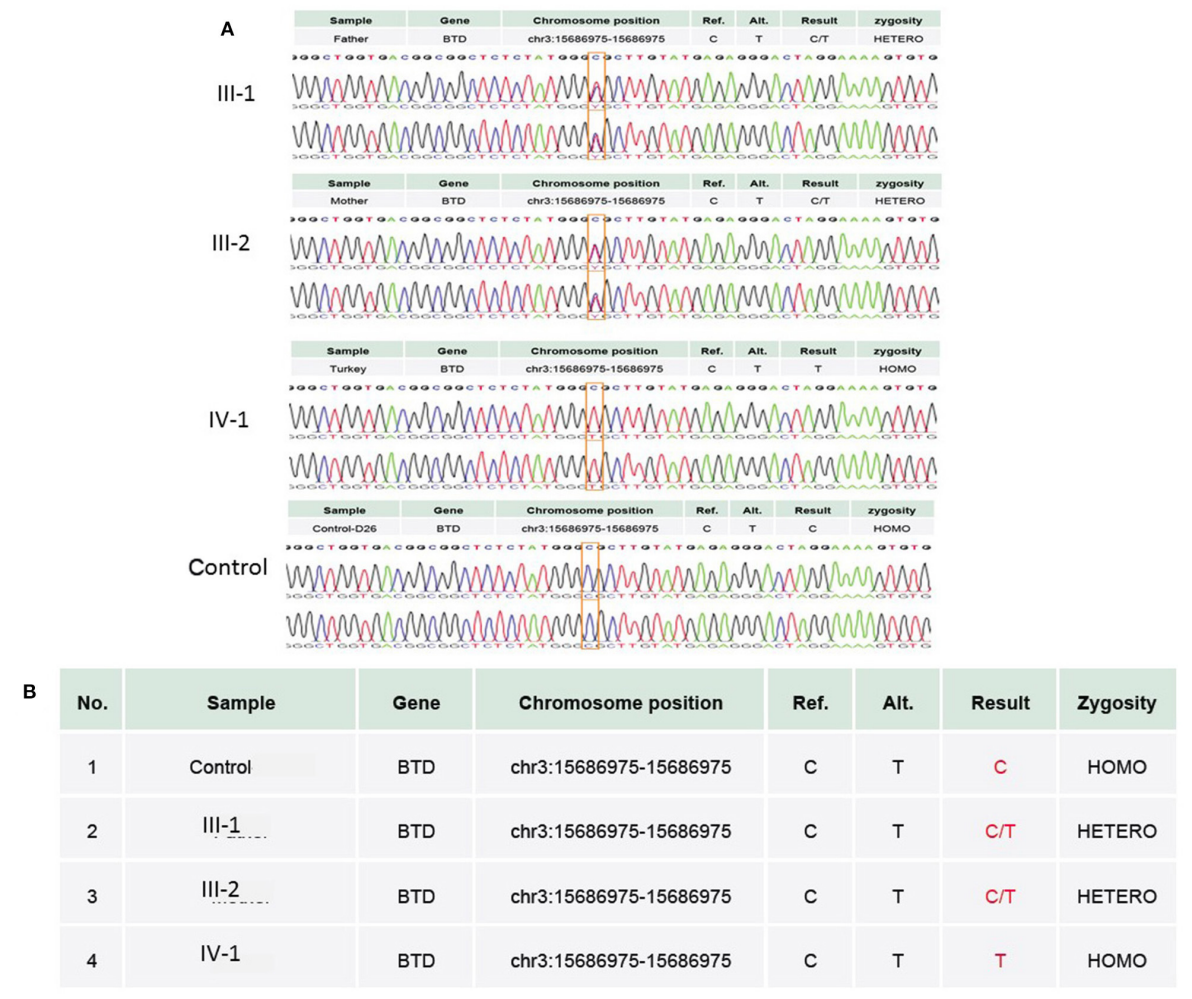
**(A)** Chromatogram of Sanger sequence analysis showing that in III-1 and III-2, both parents are the heterozygous C/T normal carrier, while IV-1 is the affected member of the family showing a sequence variation c.1612C>T, p.Arg538Cys in the *BTD* gene. **(B)** Both parents in III-1 and III-2 are heterozygous, while proband IV-1 has a T/T allele at the missense variant segregated from both parents, whereas the wild-type control has a C/C allele at the chromosome position chr3:15686975C>T.

WES results showed missense sequence variation c.1612C>T in the *BTD* gene. The missense variant NM_001370658.1 (BTD):c.1612C>T (p.Arg538Cys) causes the same amino acid change as a previously established pathogenic variant as shown in Figures 4A,B. The p.Arg538Cys variant is observed in 13/108,856 (0.0119%) alleles from individuals of gnomAD non-Finnish European background in gnomAD. The p.Arg538Cys variant is novel (not in any individuals) in the 1 kG database identified so far. Biotinidase deficiency presenting with a clinical picture mimicking NMOSD was not reported before in the Middle East region, especially in Saudi Arabia. Only few cases in the literature discussed this topic. In this study, we discuss the clinical and genetic presentation of two pediatric cases from different families having similar phenotypes.

### Protein Structure

There is a major physicochemical difference between arginine and cysteine that likely affects secondary protein structure because these residues differ in polarity, charge, size, and/or other properties. Six variants within six amino acid positions of the p.Arg538Cys variant have been shown to be pathogenic, while none have been shown to be benign. The p.Arg538Cys missense variant is classified as deleterious by both SIFT and PolyPhen-2. The c.1612C>T nucleotide in *BTD* is predicted by GERP++ and PhyloP to be conserved in 100 vertebrates. For these reasons, this variant was classified as pathogenic.

**Figure 5:**
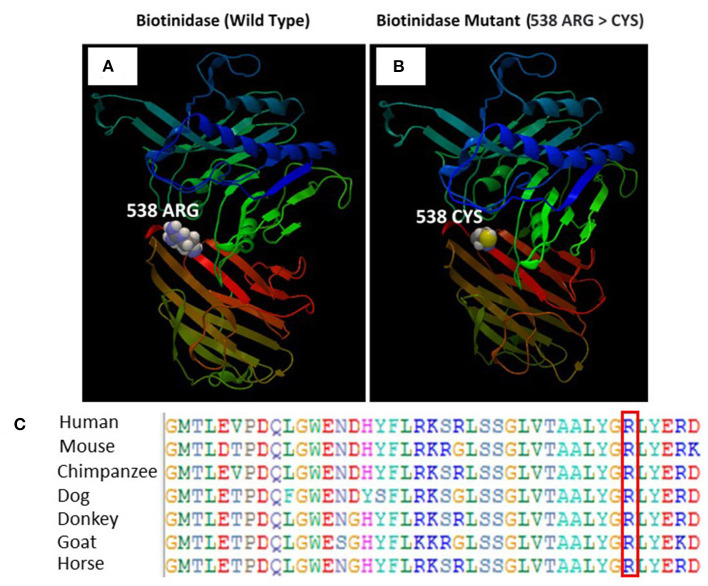
**(A)** Illustration of the wild-type and mutant structures of BTD and the prediction for the position of p.Arg538Cys. **(B)** The 3D structures of the wild-type and p.Arg538Cys mutant BTD proteins were designed using the SWISS-MODEL homology modeling platform. The amino acid Arg at position 538 in the wild-type BTD and Cys at position 538 in the mutant BTD are shown as space-filling [(Calotte or Corey, Pauling, and Koltun (CPK)] models. **(C)** The representation of the alignment of different species was performed to highlight the strong conservation of variants at p.Arg538Cys.

The SWISS-MODEL homology modeling server was used to model both wild-type and mutant protein structures for the position of p.Arg538Cys of the *BTD* gene (Figures 5A,B). Structural damage of the BTD protein is predicted because the substitution of CYS at amino acid position 538 replaces a buried charged residue (ARG, RSA 0.0%) with an uncharged residue (CYS). However, no disulfide bond disruption, no buried Pro, no collision, no buried hydrophile, no buried charge, no altered secondary structure, no buried charge change, no false phi/psi, no buried Gly, no buried H-bond disruption, no buried salt bridge disruption, no altered cavity, no buried or exposed switch, no replaced Cis-Pro, and no Gly in a bend were observed in the mutant BTD. In addition, sequencing alignment of different species was performed, and the strong conservation of the two variants in p.Arg538Cys was highlighted by downloading the data of different proteins from the Ensembl Gene Browser (https://m.ensembl.org/index.html) and by performing the sequencing alignment using the BioEdit software (Figure 5C).

## Discussion

Our two patients represent a rare form of biotinidase deficiency that clinically and radiologically mimics NMOSD. No similar cases with similar presentation have been reported in our region in Saudi Arabia.

Biotinidase deficiency is a treatable neurometabolic disease. Typically, the disease is characterized by seizures, dermatitis, hypotension, respiratory problems, and visual and auditory problems. The early-onset form usually occurs in childhood, i.e., under the age of 10. In adolescence or the late-onset form, myelopathy and visual problems may occur. Fever or concurrent infections may promote the onset of the disease. Seizures and hypotension are the most commonly reported manifestations of biotinidase deficiency. Seizures have been reported as infantile spasms, generalized epilepsy, and myoclonic seizures. Hypotonia and motor delays may be seen, especially before the age of 2 years. Symptomatic children may have apnea and difficulty breathing and eventually require a tracheostomy. Sensorineural hearing loss and optic atrophy have been reported. A low biotinidase enzyme level supports the diagnosis. WES is recommended to confirm the presence of *BTD* sequence variation.

NMOSD is an inflammatory CNS disorder usually associated with AQP4 antibodies. Classically, the inflammatory changes affect specific areas of the brain, spinal cord, and optic nerves. The disease is usually progressive, and the attacks are often severe. AQP4 is a water channel protein found in the gray matter of the spinal cord and in the periventricular and periaqueductal regions. NMOSD occurs in different age groups, in children and adults. It results in impairment of the gray matter of the central spinal cord, which shows up as T2 hyperintensity on MRI of the spine. The lesions may also involve the hypothalamus, medulla, and regions of high AQP4 expression such as the periaqueductal region. The presence of AQP4 antibodies is diagnostic of this disease among the characteristic MRI abnormalities of the brain and spine. Optic neuritis and visual disturbances develop in most patients as the disease progresses. Despite aggressive immunotherapy, the prognosis of NMOSD is poor. Recurrent attacks lead to functional deterioration due to motor, sensory, and visual deficits. Mortality rates may be as high as 50% in some studies.

Visual and sensorimotor deficits along with radiographic abnormalities on MRI of the brain and spine resembling NMOSD, along with negative AQP4 antibodies and poor response to immunotherapy, should prompt the clinician to seek alternative diagnoses, especially in children and in communities with high consanguinity. In addition to NMOSD, mitochondrial disease, multiple sclerosis, transverse myelitis, and brainstem encephalitis may also present as biotinidase deficiency.

Bottin et al. (8) reported a similar presentation in an adult patient with an initial diagnosis of NMOSD. A similar age group with myelopathy and visual and hearing symptoms was reported by Cabasson et al. (9) and Girard et al. (10) with clinical and radiological improvement after biotin supplementation. A delay in diagnosis was reported by Ferreira et al. (11). A 36-year-old woman did not experience significant improvement despite biotin supplementation because her symptoms began 20 years ago with optic atrophy and motor weakness.

Discontinuation of biotin could lead to clinical relapse, and in some cases, brain MRI could be reminiscent of NMOSD. Bilge and Yevgi (12) reported a 20-year-old patient with optic atrophy, hearing loss, and transverse myelitis who was found to have severe biotinidase deficiency and whose MRI of the brain resembled that of NMOSD.

Supplementation with biotin should be given for months before noticeable improvement occurs in some cases (13). In cases with biotinidase deficiency mimicking NMOSD, immunotherapy attempts were made in several case reports before a definitive diagnosis was made. No clinical benefit was seen in these trials. However, a few cases documented some improvement in symptoms of optic neuritis in biotinidase deficiency when steroids were administered in combination with biotin (14).

Recently, five novel mutations and one heterozygous linkage for the c.250-1G>C and c.878dupT variants were discovered in eight symptomatic patients from China, resulting in decreased protein expression due to structural damage and affecting BTD enzyme activity (15, 16). In the present study, using homology modeling of BTD, we confirmed that replacement of ARG with CYS is deleterious and results in a non-functional BTD enzyme.

Biotinidase deficiency can be detected early by newborn screening. The lower the enzyme activity, the more likely the severity of symptoms and the need for long-term biotin therapy. However, some cases may be missed by newborn screening or simply if it was not done. Severe biotinidase deficiency is documented when enzyme activity is <10%. Partial biotinidase deficiency is seen in individuals with enzyme activity between 10 and 30%.

Similar to previously reported cases, immunotherapy did not improve the situation in our patients. NMOSD and biotinidase deficiency have similar clinical and radiological features, which may lead to initial misdiagnosis in both cases. After biotin supplementation, vision improved dramatically in both of our patients. Their breathing and motor function also slowly improved.

Early detection and treatment could prevent further deterioration and permanent damage. Free biotin supplementation should be continued for life in those with severe deficiency. Doses of biotin range from 5 mg/day to 20 mg/kg/day. In acute cases, doses of up to 300 mg/day of biotin have been reported (8).

## Conclusion

Children who present with neuromyelitis optica with negative AQP4-Abs should be evaluated for biotinidase deficiency. In such cases, we recommend adding biotinidase enzyme activity determination and WES to the testing algorithm. Early diagnosis and early initiation of biotin treatment will prevent further clinical deterioration. Family screening is of great importance in identifying sequence variations in the *BTD* gene in affected individuals and helps in timely treatment.

## Materials and Methods

### Sample Collection

Saudi families with fever followed by lower limb weakness were recruited for a detailed study. The pedigree was carefully constructed by interviewing all family members. Peripheral blood samples were collected in EDTA tubes from all available family members. Genomic DNA was extracted using QIAamp genomic DNA extraction or similar kits according to the manufacturer's protocols (https://www.qiagen.com/pk/products/top-sellers/qiaamp-dna-minikit/#orderinginformation). Quantification of genomic DNA was performed using a NanoDrop spectrophotometer (https://www.thermofisher.com/order/catalogue/product/ND-LITE-PR), and visualization was performed using the SYBR Safe dye (Thermo Fisher, USA) by running a 1% agarose horizontal gel electrophoresis apparatus. Written informed consent was obtained from all participants before the start of the study. This study was approved by the local ethics committee of King Abdulaziz University and complied with all the guidelines of the Helsinki Declaration of 2013.

### WES

RNA capture baits against ~60 Mb of the human exome (targeting > 99% of the regions in the CCDS, RefSeq, and GENCODE databases) are used to enrich regions of interest from fragmented genomic DNA using Agilent's SureSelect Human All Exon V6 Kit, as explained previously (17). The generated library is sequenced on an Illumina platform to achieve an average depth of coverage of ~100×. Typically, ~97% of target bases are covered >10×. After WES, FASTQ files were converted to BAM, and then BAM files were converted to variant call format (vcf), obtaining a total of 113,830 variants. These variants were used to identify mutations that may lead to the disease based on the frequency of novel/rare variants (MAF+0.01%), function (predicted damage by PolyPhen/SIFT), homozygous/heterozygous state, genomic position, pathogenicity, protein action, and previous associations with disease-related phenotype. We used several filters and bioinformatics tools. The following databases and *in silico* algorithms were used to annotate and assess the impact of the variant in the context of human disease: 1,000 Genomes, gnomAD, ClinVar, OMIM, dbSNP, NCBI RefSeq Genes, ExAC Gene Constraints, VS-SIFT, VS-PolyPhen-2, PhyloP, GERP++, GeneSplicer, MaxEntScan, NNSplice, and PWM Splice Predictor. Analysis was performed using the HGVS nomenclature (www.hgvs.org/mutnomen) as implemented by the VarSeq transcript annotation algorithm.

The sequenced reads are aligned to GRGh37 using BWA-mem. Variants are classified and annotated using the Golden Helix VarSeq analysis workflow, which implements ACMG guidelines. An in-house bioinformatics pipeline includes base calling, alignment of reads to GRCh37/hg19 genome assembly, primary filtering of low-quality reads and likely artifacts, and subsequent annotation of variants. All disease-causing variants reported in HGMD^®^, in ClinVar, or in CentoMD^®^ are considered, as well as all variants with a minor allele frequency (MAF) of <1% in the gnomAD database. The evaluation focuses on the coding exons as well as the flanking ±20 intronic bases. All relevant inheritance patterns are considered. In addition, the provided family history information and clinical data will be used to evaluate any identified variants. All identified variants will be evaluated for pathogenicity and causality. All variants associated with the patients' disease, excluding benign or likely benign variants, will be reported. Lower-quality single-nucleotide or deletion–insertion variants will therefore be confirmed by Sanger sequencing analysis.

### Sanger Sequencing

Further WES results were validated with the Sanger sequencing technique using the targeted primers of the reported sequence variation in the *BTD* gene. Sanger sequencing was performed as previously explained by Naseer et al. (18). This variation was not identified even in 100 unrelated healthy individuals in the population. Both parents were heterozygous carriers, and the proband had a homozygous sequence variation.

### Generation of a 3D BTD Homology Model

The homology model of the BTD protein was constructed using the automated homology modeling platform SWISS-MODEL (19). The FASTA sequence of BTD isoform 1 was downloaded from the UniProt Knowledgebase (UniProt ID: P43251-1), corresponding to a 543-amino-acid transcript (Ensembl ID: ENSG00000169814) (20). A template search using BLAST and HHblits was performed against the SWISS-MODEL template library (SMTL, last update: 2021-12-22, last included PDB release: 2021-12-17) as previously described (21). The target sequence was aligned to the primary amino acid sequence included in the SMTL (22). An initial HHblits profile was generated (23, 24) and aligned with all profiles in the SMTL. A total of 265 templates were identified. The BTD homology model was built using pantetheinase or vanin-1 (PDB ID: 4CYF) based on target and template alignment using ProMod3 (25). The quality of the global model and the quality of the individual residue models were assessed using the QMEAN scoring function (26).

### BTD Mutation Analysis Using Missense 3D

The effects of substituting Arg with Cys on the BTD homology model were investigated by examining the structural features available in the Missense 3D algorithm (21, 27), such as buried charge change, buried proline insertion, disulfide bond breakage, buried hydrophilic residue insertion, buried salt bridge break, buried charge insertion, buried glycine replacement, collision, buried H-bond break, secondary structure change, allowed phi/psi, buried charge replacement, cavity change, buried/exposed change, and glycine in a bend (21).

## Data Availability Statement

The original contributions presented in the study are included in the article/supplementary material, further inquiries can be directed to the corresponding author/s.

## Ethics Statement

The studies involving human participants were reviewed and approved by Center of Excellence in Genomic Medicine Research King Abdulaziz University Ethical Committee. Written informed consent to participate in this study was provided by the participants' legal guardian/next of kin. Written informed consent was obtained from the individual(s), and minor(s)' legal guardian/next of kin, for the publication of any potentially identifiable images or data included in this article.

## Author Contributions

MN and AA designed the experiments. PP and MN conducted the experiments. PP, AA, OM, and MN analyzed the data. OM and MN wrote the manuscript. PP, OM, and MN finally revised the manuscript. All authors contributed to the editing of the manuscript and the scientific discussions.

## Funding

This research work was funded by the Institutional Fund Project Under Grant No. IFPRC-101-662-2020.

## Conflict of Interest

The authors declare that the research was conducted in the absence of any commercial or financial relationships that could be construed as a potential conflict of interest.

## Publisher's Note

All claims expressed in this article are solely those of the authors and do not necessarily represent those of their affiliated organizations, or those of the publisher, the editors and the reviewers. Any product that may be evaluated in this article, or claim that may be made by its manufacturer, is not guaranteed or endorsed by the publisher.
